# Supplementary material for: Causal connections between socioeconomic disparities and COVID-19 in the USA
Source: Sci Rep. 2022 Sep 22;12:15827. doi: 10.1038/s41598-022-18725-4 (PMC9499932; doi:10.1038/s41598-022-18725-4)
Supplement: Supplementary file 1 — Supplementary Information. [file 41598_2022_18725_MOESM1_ESM.pdf]

Causal connections between socioeconomic disparities and COVID-19 in the USA

Tannista Banerjee, Ayan Paul, Vishak Srikanth, and Inga Strümke

Confounding variables

Given the complexity of the problems that we address the relative difficulty in engineering all the variables necessary to address it, there is always a possibility of the existence of confounding variables in the analysis. The causal Shapley value framework, as described in the Materials and Methods section, allow for the assumption of the existence of confounding variables within a group of variables that are not causally connected or are at the same level in the directed acyclic graph. To test whether assuming the presence of confounding variables change the results of our analysis we performed all the analysis for the confirmed case rate (two phases, three regions and six causal ordering) assuming the presence of confounding variables in all the groups. The results of our analysis is shown in Figure S1 and Figure S2. It can be seen that assuming the presence of confounding variables do not change the final result of the analysis at all. Hence, we can say with confidence that we safely ignore the presence of confounding variables. That being said, one can argue that variables like the average level of education in a county are highly correlated with several of the socioeconomic metrics that we consider and maybe it can replace one of the metrics that we consider here. However, since we test the causal connections between the variables under a certain hypothesis represented by the directed acyclic graphs, studying the details of all possible hypothesis is beyond the scope of our work and we would like to motivate future works to consider a different set of variable to understand better, what the true causations in the real-world are.

Additional causal orderings

We have considered three causal orderings in the main text. To give some more possible scenarios for the causal dependence withing the socioeconomic metrics we propose three more causal ordering. We will try to motivate them here and provide the results in the figures that follow

CO#4: The fourth causal ordering is [NW, SC, [Emp, Uemp], [Inc, Lab], [Uins, Com, Pov, GI], [Den, MC, Tran]]. This causal ordering builds upon the first causal ordering CO#1, except that the proportion of senior citizens is no longer on equal footing as unemployment and employed, but moved up in the causal chain. This causal ordering takes into account the fact that senior citizens who are looking for employment may not be able to find employment due to a skills mismatch with the required skills today and senior citizens may be structurally unemployed [1,2].

CO#5: The fifth causal ordering is [NW, [SC, Emp, Uemp, Inc, Lab], [Uins, Com, Pov, GI], [Den, MC, Tran]]. This causal ordering has income per capita, the proportion of a county working in construction, service, delivery or production, unemployment, employed, and the proportion of senior citizens on equal footing. The rationale here is that income per capita and the proportion of a county working in construction, service, delivery or production are on equal footing with unemployment and employment since construction, service, delivery, or production jobs are (a) more likely to be temporary (b) pay low wages and counties with people working in those job profiles tend to have fewer permanent jobs and a higher unemployment rate due to the decline of manufacturing in the last half century [3].

CO#6: The sixth causal ordering is [SC, NW, [Emp, Uemp], [Inc, Lab], [Uins, Com, Pov, GI], [Den, MC, Tran]]. This causal ordering has the proportion of senior citizens causing the proportion of non-whites, and is otherwise the same as the first causal ordering CO#1. Since senior citizens are less likely to be non-white, a higher fraction of senior citizens causes there to be a lower proportion of non-whites [4].

The results obtained by assuming these causal ordering between the socioeconomic metrics can be found in Figure S3 and Figure S4. The results from these causal orderings are quite similar to the ones obtained from the CO#1, CO#2 and CO#3, pointing to the same sets of variables as the most causally connected ones in Phase I. In Phase II we see the diminished impact of the socioeconomic metrics in all regions except for the west coast states.

Causal analyses for death rates

The analysis in the main paper focuses only on understanding the causal connections between the socioeconomic metrics and the confirmed case rate. A similar analysis can be performed using the death rate as the endogenous variable. We show the results of this analysis in Figure S5 to Figure S8. We see that the landscape of the causal dependence is a bit different for the

	Den	NW	Pov	Inc	Uemp	Uins	Emp	Lab	Tran	MC	SC	GI	Com
mean	273.14	23.75	15.11	28071.83	5.29	9.64	55.11	47.53	15.20	23.85	18.79	0.45	46.79
std	1797.52	20.22	6.33	6782.95	2.65	5.11	8.27	7.32	6.55	5.86	4.66	0.04	6.38
min	0.04	0.00	2.40	10388.00	0.00	0.00	14.70	14.20	0.00	5.10	3.20	0.30	22.00
25%	16.52	7.52	10.60	23613.75	3.60	5.80	49.80	43.40	11.91	19.80	15.80	0.42	42.90
50%	44.84	16.26	14.20	27301.50	4.90	8.70	55.70	48.30	14.03	23.70	18.45	0.44	47.20
75%	118.55	35.56	18.48	31245.25	6.50	12.10	61.20	52.40	16.76	27.60	21.20	0.47	51.00
max	71874.13	99.31	55.50	76592.00	27.20	46.30	80.00	78.70	92.43	52.60	56.70	0.71	66.20

Table S1. *Various statistics of the data collected from the US Census Bureau and Centers for Disease Control and Prevention. Den: Population Density, NW: Non-White, Pov: Poverty, Inc: Income, Uemp: Unemployment, Uins: Uninsured, Emp: Employed, Lab: Labor, Tran: Transit, MC: Mean Commute, SC: Senior citizen, GI: Gini Index, Com.: Comorbidities. Density is measured per sq. km, Income in US dollars, Transit in minutes and the rest are percentages. For details on the data sources and curation please refer to the Methods section in the main text. Details of data curation for Comorbidities can be found in Ref. [5].*

death rates as compared with the confirmed case rates. There seems to be a higher causal connection between a larger set of socioeconomic metrics and death rates in Phase I. The top three causations are Density, Non-White and Mean Commute for the East Coast region irrespective of the causal ordering assumed. For the West Coast region the Senior Citizen metric seems to almost always appear on the top. For the Southern States region, Non-White and Density are always the top two causations. In Phase II the causal connections are significantly diluted between the exogenous and endogenous variables in all regions except that we still see some causal connection between Senior Citizen and death rates in the West Coast region and as always it is anti-correlated pointing to the fact that the death rates are higher in the counties in which the fraction of senior citizen is lower and the overall population is younger.

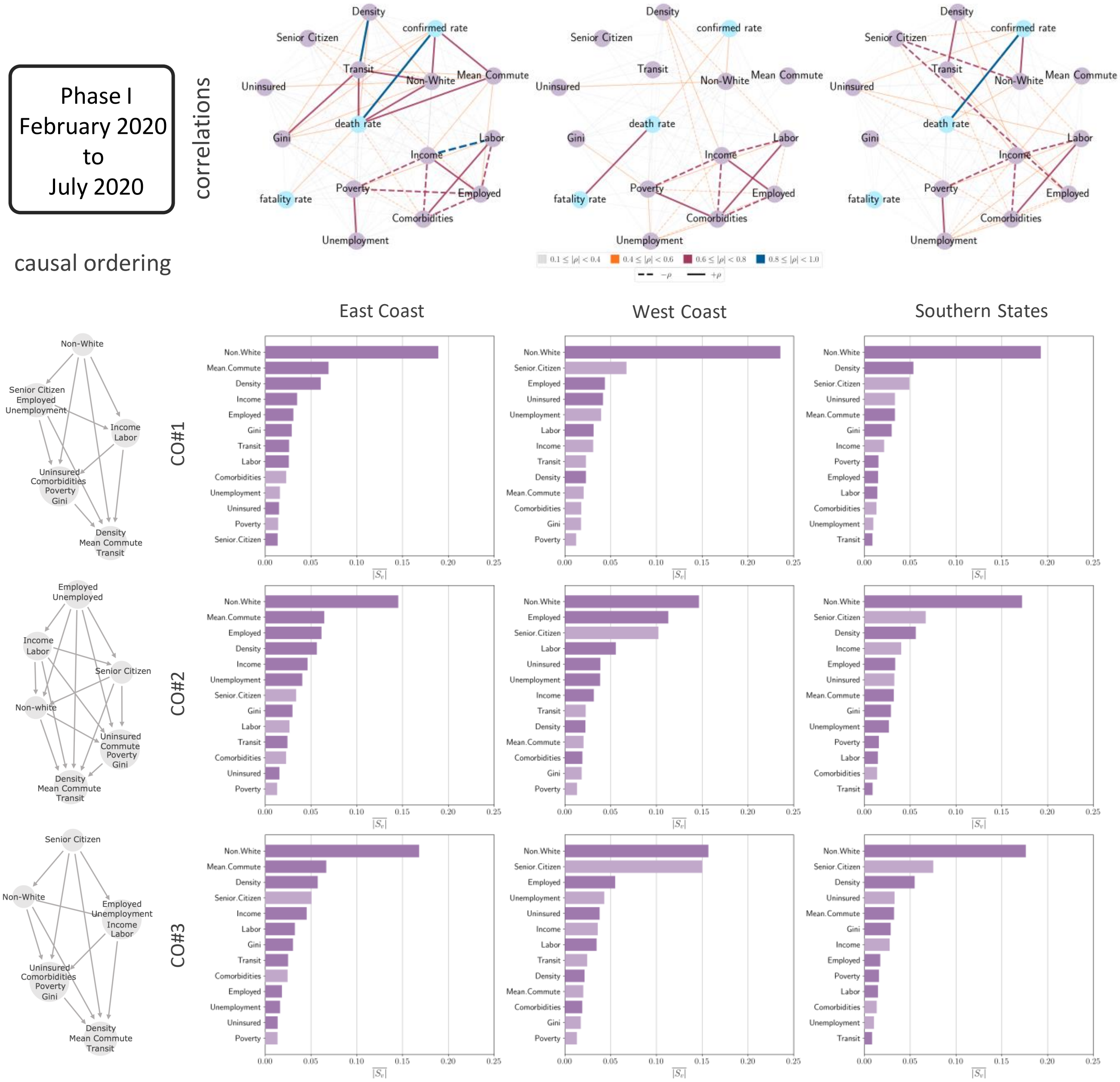

Figure S1. This analysis is performed assuming the presence of confounding variables in each group of variables in the causal ordering. For details please see text. *The causal connection between the socioeconomic metrics that we consider in this work and confirmed case rates in various regions of the USA from February 2020 to July 2020. The states included in the East Coast region are District of Columbia, New Jersey, Rhode Island, Massachusetts, Connecticut, Maryland, Delaware and New York. The ones in the West Coast region are California, Oregon and Washington. The states in the Southern States region are Alabama, Arkansas, Florida, Georgia, Kentucky, Louisiana, Mississippi, North Carolina, Oklahoma, South Carolina, Tennessee, Texas, Virginia and West Virginia. The networks in the top row show the correlations between the different metrics in the three regions. The graphs in the left column of the plot show the three partial causal orderings that we consider in this analysis. Each panel of the array of bar plots show the hierarchy of the metrics in terms of mean absolute causal SHAP values,* $\overline{|S_v|}$, *for a particular region and causal ordering. The lighter shaded bars indicate a negative effect of the metric on the confirmed case rates while the darker bars indicate a positive effect.*

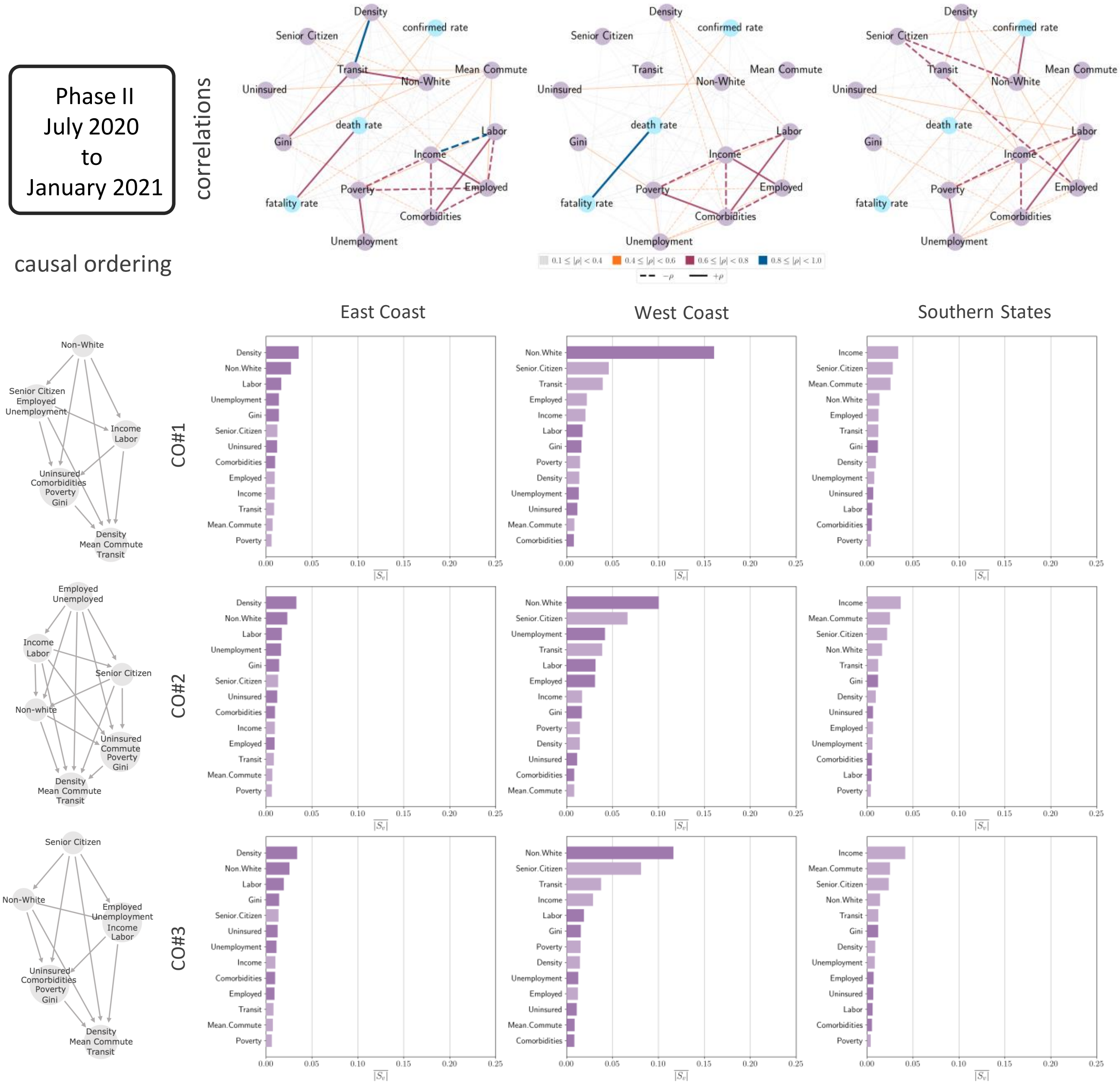

Figure S2. The analysis is performed assuming the presence of confounding variables in each group of variables in the causal ordering. For details please see text. *The causal connection between the socioeconomic metrics that we consider in this work and confirmed case rates in various regions of the USA from July 2020 to January 2021. The states included in the East Coast region are District of Columbia, New Jersey, Rhode Island, Massachusetts, Connecticut, Maryland, Delaware and New York. The ones in the West Coast region are California, Oregon and Washington. The states in the Southern States region are Alabama, Arkansas, Florida, Georgia, Kentucky, Louisiana, Mississippi, North Carolina, Oklahoma, South Carolina, Tennessee, Texas, Virginia and West Virginia. The networks in the top row show the correlations between the different metrics in the three regions. The graphs in the left column of the plot show the three partial causal orderings that we consider in this analysis. Each panel of the array of bar plots show the hierarchy of the metrics in terms of mean absolute causal SHAP values,* $\overline{|S_v|}$, *for a particular region and causal ordering. The lighter shaded bars indicate a negative effect of the metric on the confirmed case rates while the darker bars indicate a positive effect.*

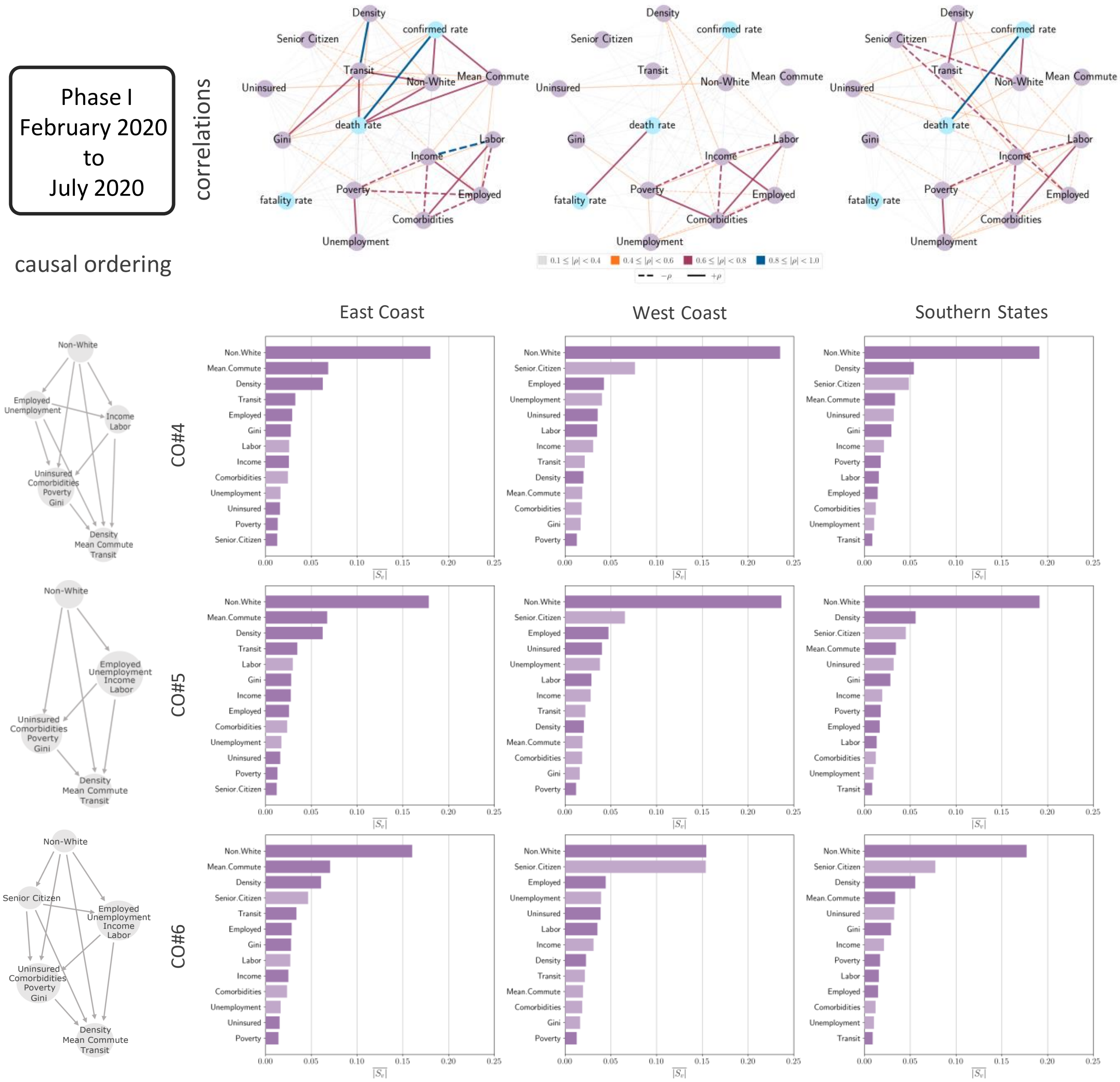

Figure S3. *The causal connection between the socioeconomic metrics that we consider in this work and confirmed case rates in various regions of the USA from February 2020 to July 2020. The states included in the East Coast region are District of Columbia, New Jersey, Rhode Island, Massachusetts, Connecticut, Maryland, Delaware and New York. The ones in the West Coast region are California, Oregon and Washington. The states in the Southern States region are Alabama, Arkansas, Florida, Georgia, Kentucky, Louisiana, Mississippi, North Carolina, Oklahoma, South Carolina, Tennessee, Texas, Virginia and West Virginia. The networks in the top row show the correlations between the different metrics in the three regions. The graphs in the left column of the plot show the three partial causal orderings that we consider in this analysis. Each panel of the array of bar plots show the hierarchy of the metrics in terms of mean absolute causal SHAP values,* $\overline{|S_v|}$, *for a particular region and causal ordering. The lighter shaded bars indicate a negative effect of the metric on the confirmed case rates while the darker bars indicate a positive effect.*

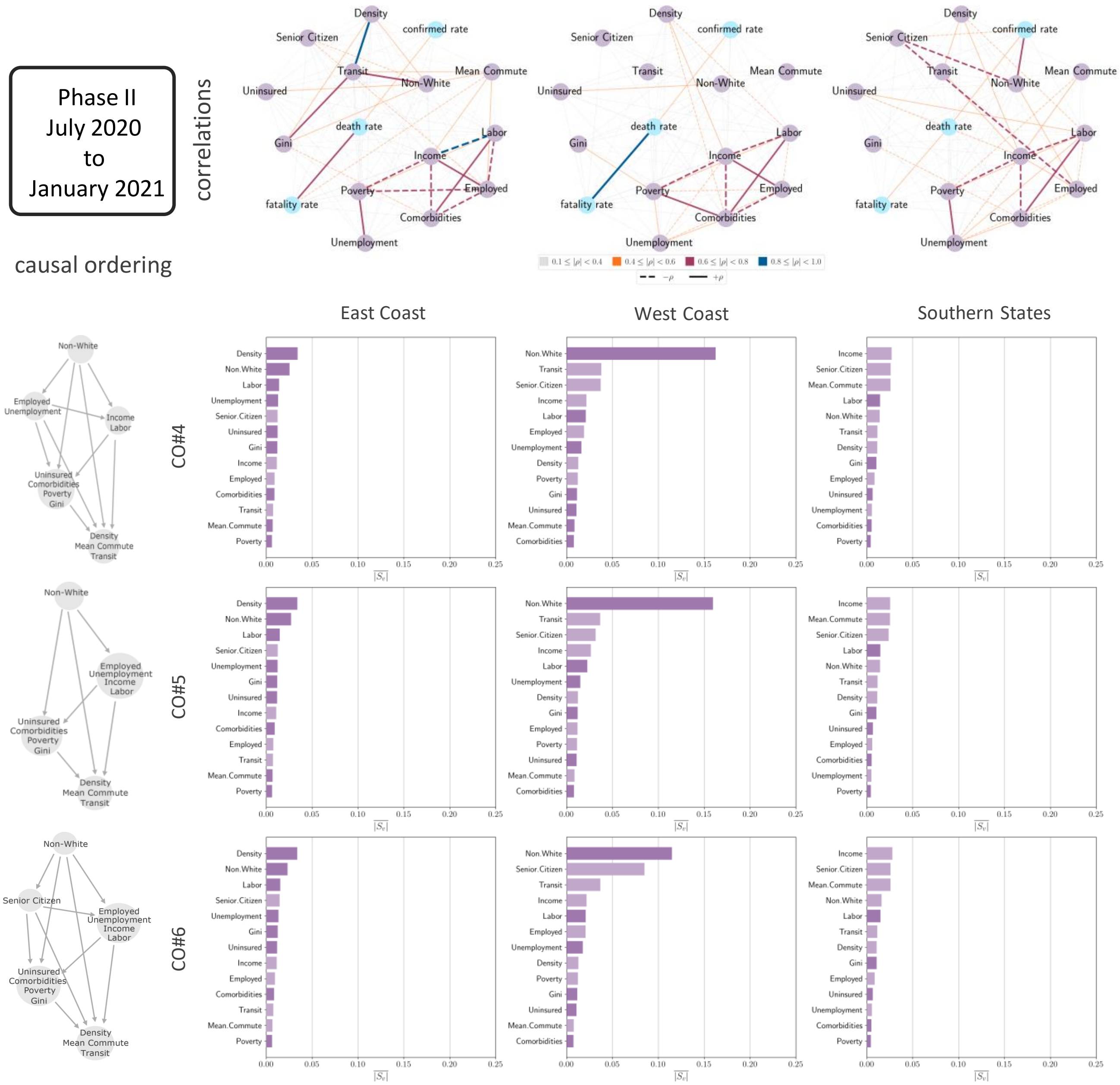

Figure S4. *The causal connection between the socioeconomic metrics that we consider in this work and confirmed case rates in various regions of the USA from July 2020 to January 2020. The states included in the East Coast region are District of Columbia, New Jersey, Rhode Island, Massachusetts, Connecticut, Maryland, Delaware and New York. The ones in the West Coast region are California, Oregon and Washington. The states in the Southern States region are Alabama, Arkansas, Florida, Georgia, Kentucky, Louisiana, Mississippi, North Carolina, Oklahoma, South Carolina, Tennessee, Texas, Virginia and West Virginia. The networks in the top row show the correlations between the different metrics in the three regions. The graphs in the left column of the plot show the three partial causal orderings that we consider in this analysis. Each panel of the array of bar plots show the hierarchy of the metrics in terms of mean absolute causal SHAP values,* $\overline{|S_v|}$, *for a particular region and causal ordering. The lighter shaded bars indicate a negative effect of the metric on the confirmed case rates while the darker bars indicate a positive effect.*

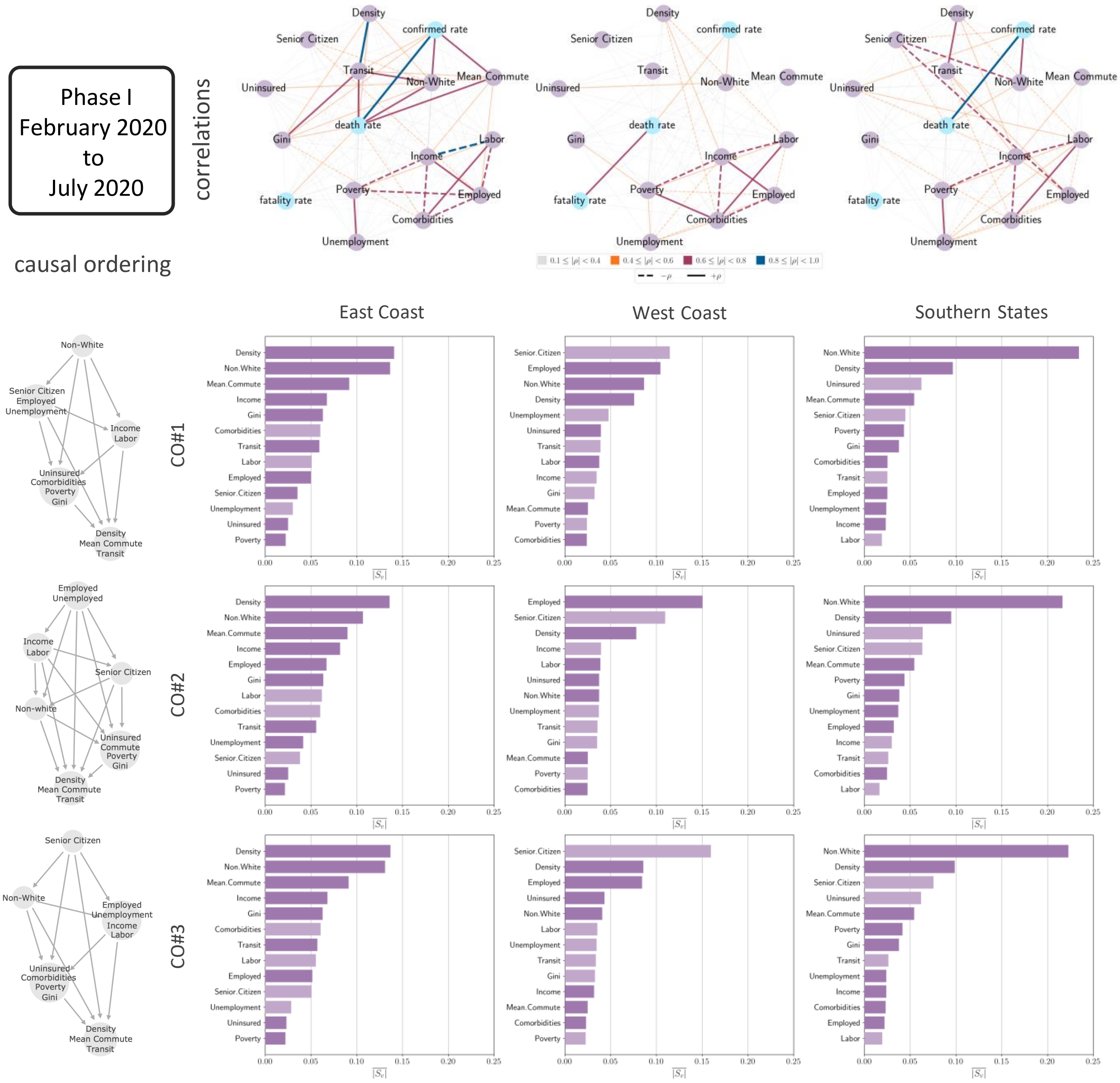

Figure S5. *The causal connection between the socioeconomic metrics that we consider in this work and death rates in various regions of the USA from February 2020 to July 2020. The states included in the East Coast region are District of Columbia, New Jersey, Rhode Island, Massachusetts, Connecticut, Maryland, Delaware and New York. The ones in the West Coast region are California, Oregon and Washington. The states in the Southern States region are Alabama, Arkansas, Florida, Georgia, Kentucky, Louisiana, Mississippi, North Carolina, Oklahoma, South Carolina, Tennessee, Texas, Virginia and West Virginia. The networks in the top row show the correlations between the different metrics in the three regions. The graphs in the left column of the plot show the three partial causal orderings that we consider in this analysis. Each panel of the array of bar plots show the hierarchy of the metrics in terms of mean absolute causal SHAP values,* $\overline{|S_v|}$, *for a particular region and causal ordering. The lighter shaded bars indicate a negative effect of the metric on the confirmed case rates while the darker bars indicate a positive effect.*

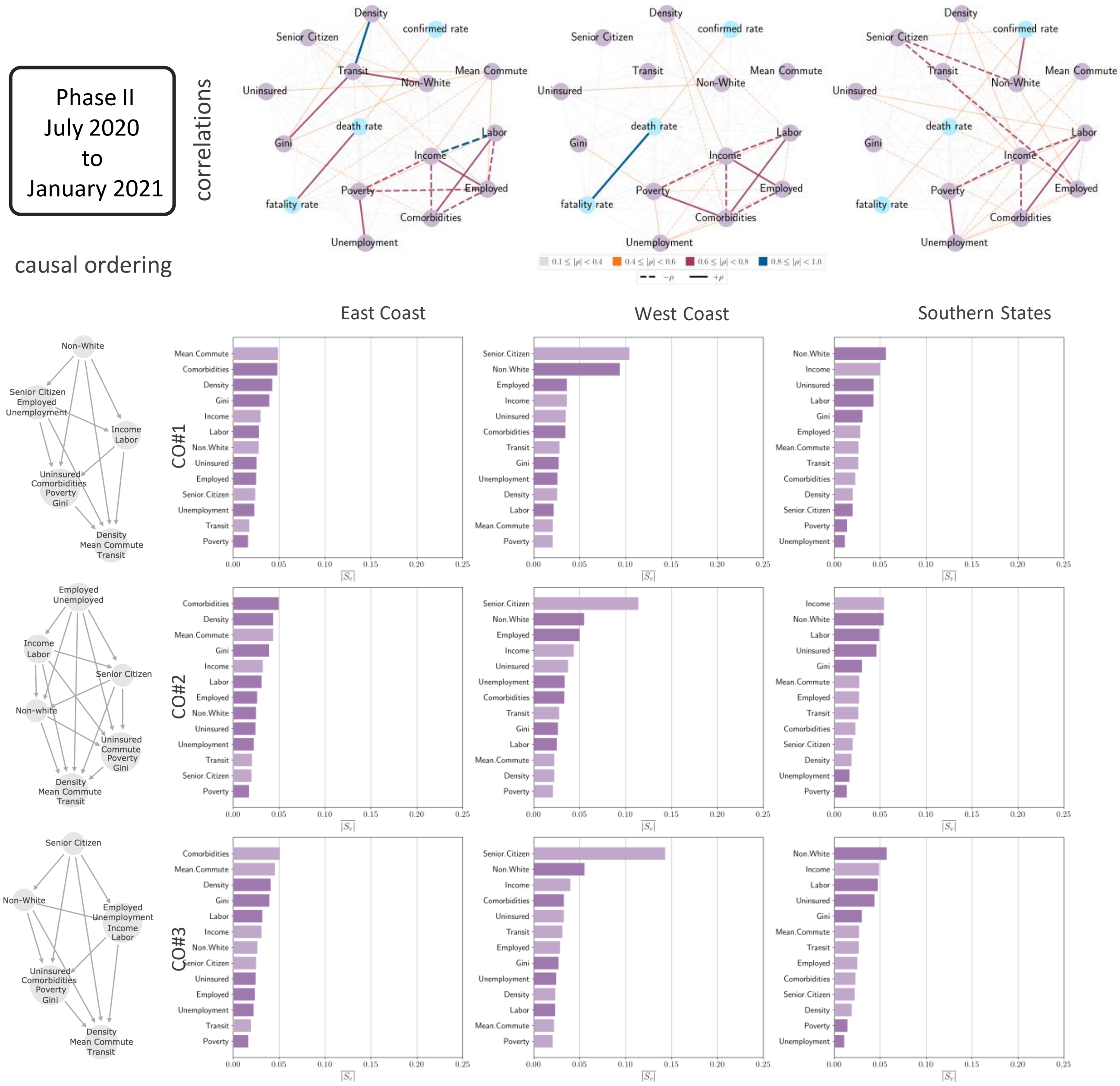

Figure S6. *The causal connection between the socioeconomic metrics that we consider in this work and death rates in various regions of the USA from July 2020 to January 2021. The states included in the East Coast region are District of Columbia, New Jersey, Rhode Island, Massachusetts, Connecticut, Maryland, Delaware and New York. The ones in the West Coast region are California, Oregon and Washington. The states in the Southern States region are Alabama, Arkansas, Florida, Georgia, Kentucky, Louisiana, Mississippi, North Carolina, Oklahoma, South Carolina, Tennessee, Texas, Virginia and West Virginia. The networks in the top row show the correlations between the different metrics in the three regions. The graphs in the left column of the plot show the three partial causal orderings that we consider in this analysis. Each panel of the array of bar plots show the hierarchy of the metrics in terms of mean absolute causal SHAP values,* $\overline{|S_v|}$, *for a particular region and causal ordering. The lighter shaded bars indicate a negative effect of the metric on the confirmed case rates while the darker bars indicate a positive effect.*

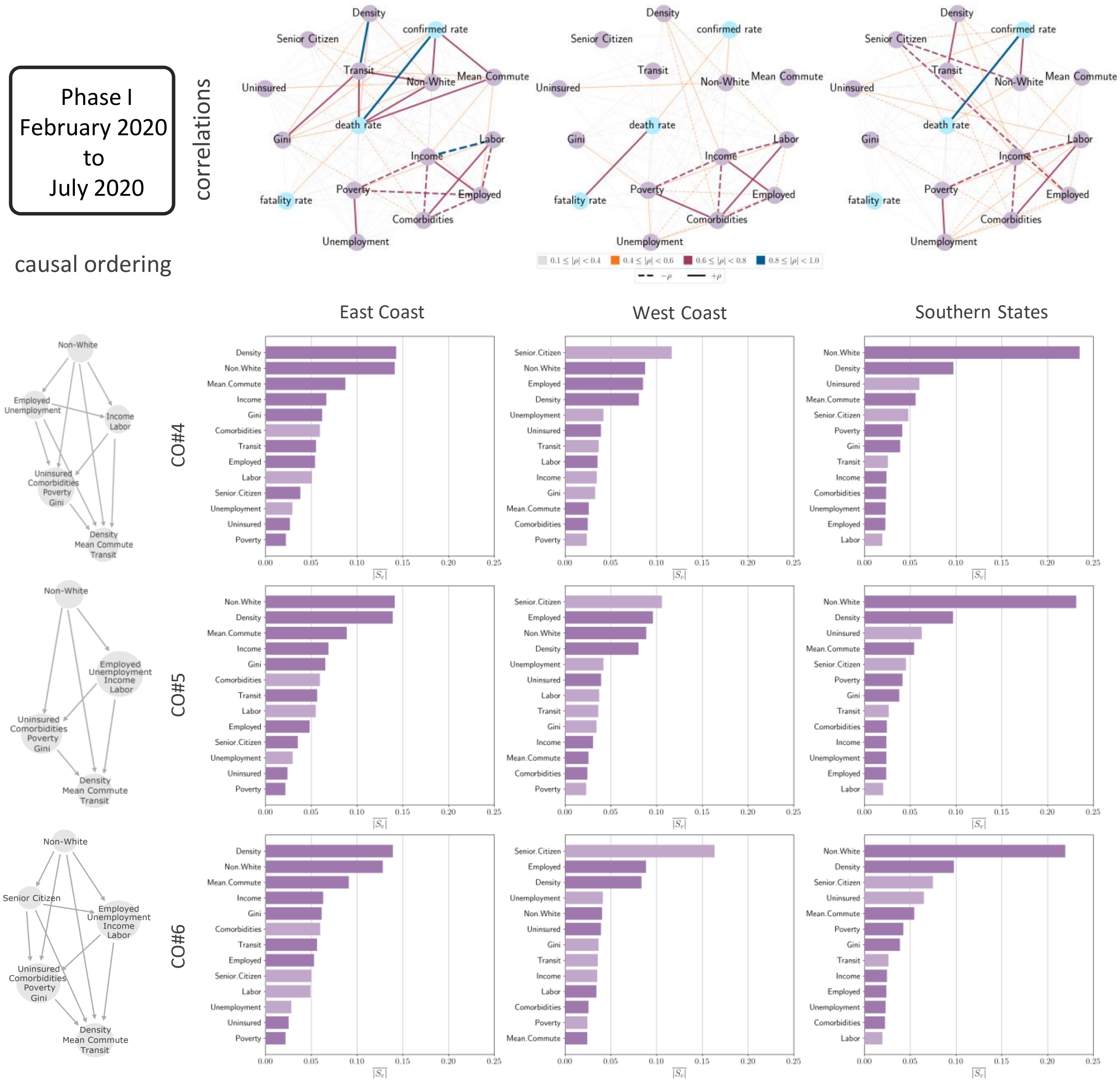

Figure S7. *The causal connection between the socioeconomic metrics that we consider in this work and death rates in various regions of the USA from February 2020 to July 2020. The states included in the East Coast region are District of Columbia, New Jersey, Rhode Island, Massachusetts, Connecticut, Maryland, Delaware and New York. The ones in the West Coast region are California, Oregon and Washington. The states in the Southern States region are Alabama, Arkansas, Florida, Georgia, Kentucky, Louisiana, Mississippi, North Carolina, Oklahoma, South Carolina, Tennessee, Texas, Virginia and West Virginia. The networks in the top row show the correlations between the different metrics in the three regions. The graphs in the left column of the plot show the three partial causal orderings that we consider in this analysis. Each panel of the array of bar plots show the hierarchy of the metrics in terms of mean absolute causal SHAP values,* $\overline{|S_v|}$, *for a particular region and causal ordering. The lighter shaded bars indicate a negative effect of the metric on the confirmed case rates while the darker bars indicate a positive effect.*

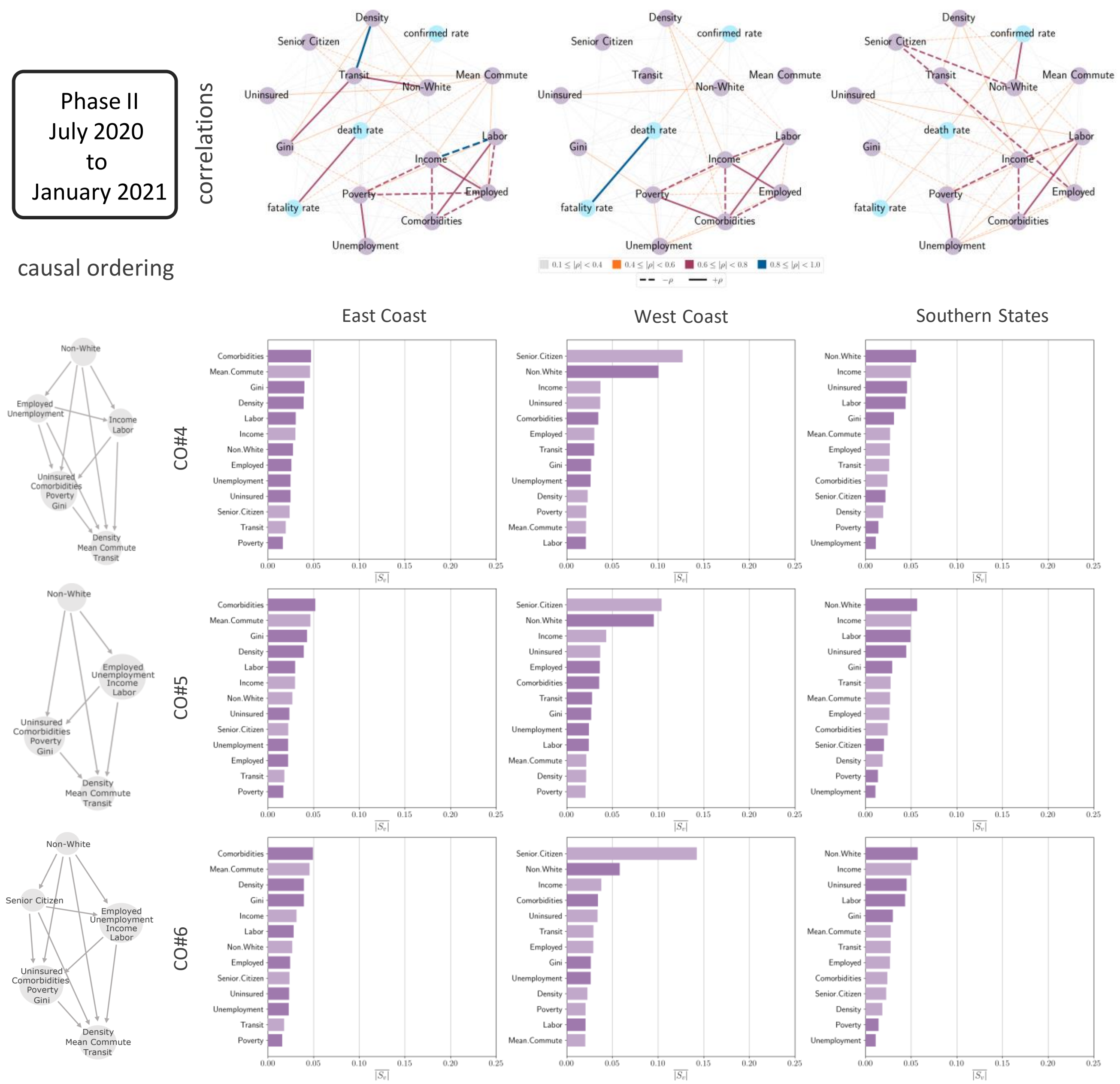

Figure S8. *The causal connection between the socioeconomic metrics that we consider in this work and death rates in various regions of the USA from July 2020 to January 2021. The states included in the East Coast region are District of Columbia, New Jersey, Rhode Island, Massachusetts, Connecticut, Maryland, Delaware and New York. The ones in the West Coast region are California, Oregon and Washington. The states in the Southern States region are Alabama, Arkansas, Florida, Georgia, Kentucky, Louisiana, Mississippi, North Carolina, Oklahoma, South Carolina, Tennessee, Texas, Virginia and West Virginia. The networks in the top row show the correlations between the different metrics in the three regions. The graphs in the left column of the plot show the three partial causal orderings that we consider in this analysis. Each panel of the array of bar plots show the hierarchy of the metrics in terms of mean absolute causal SHAP values,* $\overline{|S_v|}$, *for a particular region and causal ordering. The lighter shaded bars indicate a negative effect of the metric on the confirmed case rates while the darker bars indicate a positive effect.*

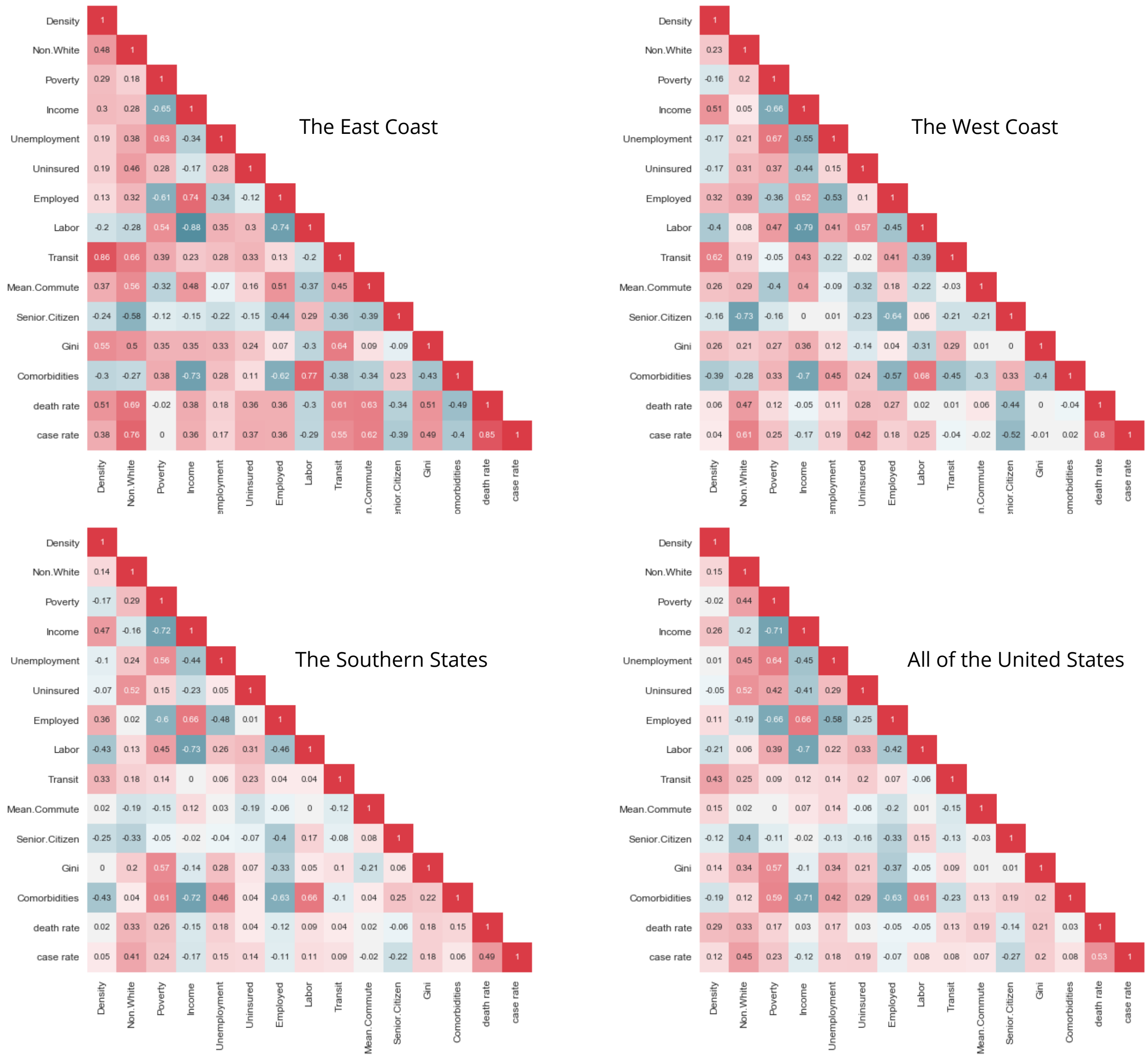

Figure S9. *Correlation. matrices for the three regions of the USA considered in this work and the one for all of the USA. for Phase I of the pandemic.*

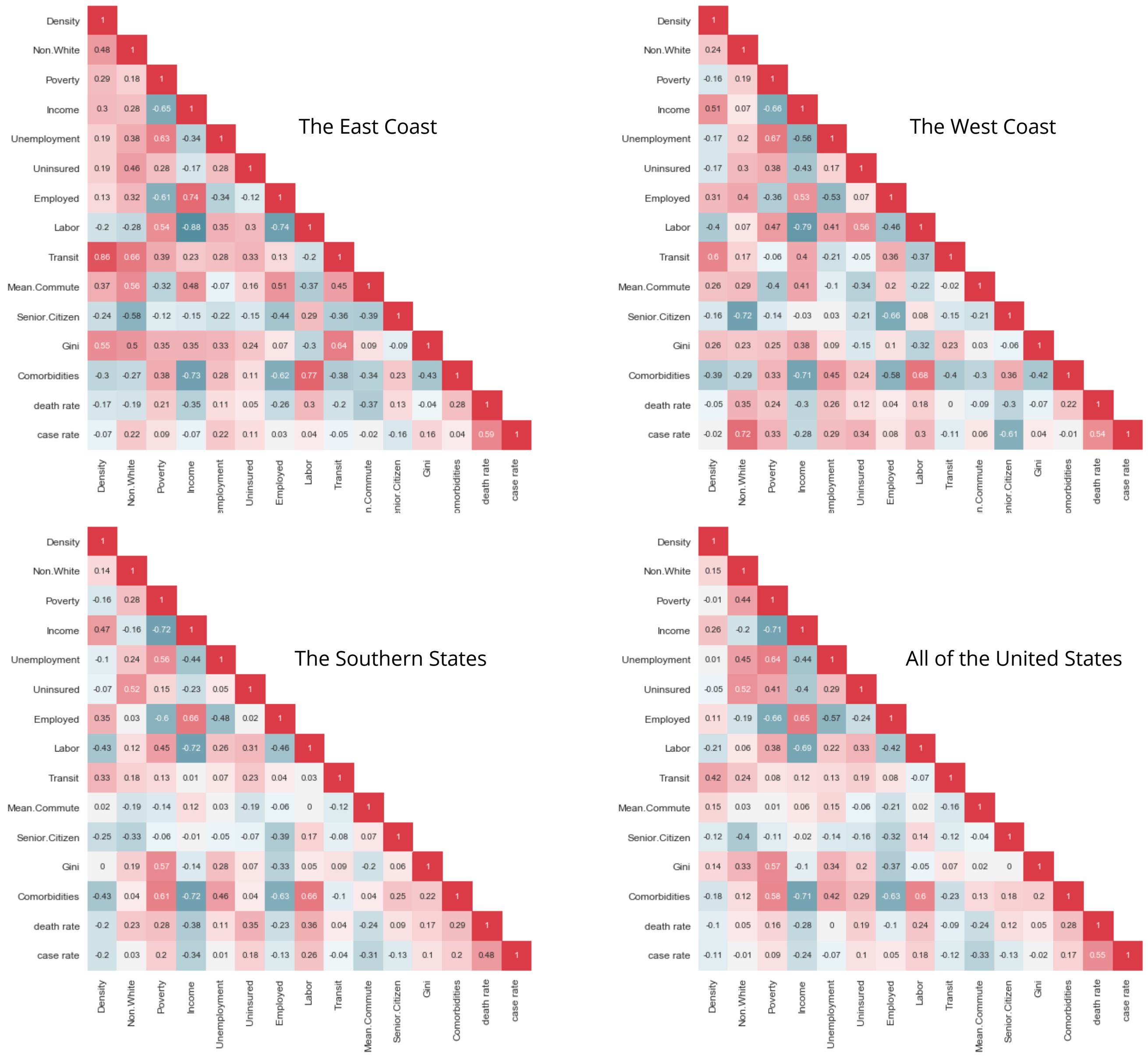

Figure S10. *Correlation. matrices for the three regions of the USA considered in this work and the one for all of the USA. for Phase II of the pandemic.*

References

1. Rust, J. & Phelan, C. How Social Security and Medicare Affect Retirement Behavior In a World of Incomplete Markets. *Econometrica* **65**, 781, DOI: 10.2307/2171940 (1997).
2. Peterson, C. L. & Murphy, G. Transition from the Labor Market: Older Workers and Retirement. *Int. J. Heal. Serv.* **40**, 609–627, DOI: 10.2190/hs.40.4.c (2010).
3. Ono, Y. & Sullivan, D. Manufacturing Plants' Use of Temporary Workers: An Analysis Using Census Microdata. *Ind. Relations: A J. Econ. Soc.* **52**, 419–443, DOI: 10.1111/irel.12018 (2013).
4. Bélanger, A. Diversity Explosion: How New Racial Demographics are Remaking America. *Can. Stud. Popul.* **43**, 166, DOI: 10.25336/p69s3v (2016).
5. Razzaghi H, L. H., Wang Y *et al.* Estimated County-Level Prevalence of Selected Underlying Medical Conditions Associated with Increased Risk for Severe COVID-19 Illness — United States, 2018. *MMWR Morb Mortal Wkly Rep.* **69**, 945–950, DOI: 10.15585/mmwr.mm6929a1 (2020).
